# Effects of an evidence-based practice education program using multifaceted interventions: a quasi-experimental study with undergraduate nursing students

**DOI:** 10.1186/s12909-019-1501-6

**Published:** 2019-03-04

**Authors:** Jeong Sook Kim, Mee Ock Gu, HeeKyung Chang

**Affiliations:** 1 Department of Nursing, Jinju Health College, 52655, 51, Uibyeong-ro, Jinju, Gyeongnam South Korea; 2 College of Nursing, Gerontological Health Research Center in Institute of Health Sciences, Gyeongsang National University, 52727, 816-15, Jinju-daero, Jinju, Gyeongnam South Korea

**Keywords:** Evidence-based practice, Simulation, Critical thinking, Nursing students

## Abstract

**Background:**

Although Evidence-Based Practice (EBP) should be introduced early on in nursing education to develop students’ independence and self-learning ability, there are few such courses for undergraduate nursing students in Korea. This study examined the effects of the EBP education program for undergraduate nursing students (EBP-EPUNS) on nursing students’ knowledge, skills, attitudes, competencies, and future use of EBP.

**Methods:**

A quasi-experimental study design with pre-test, intervention, and post-test was used. The participants were 44 nursing students (experimental: 22, control: 22). A 20-h long EBP-EPUNS consisting of 5-step EBP components was provided through 8 sessions spread across 4 weeks.

**Results:**

An independent t-test and a repeated-measures ANOVA showed that the experimental group had statistically significant higher post-test scores on EBP knowledge (*p* < 0.001), skills (*p* < 0.001), attitudes (*p* < 0.001), competencies (*p* < 0.001), future use of EBP (*p* = 0.001), and critical thinking (*p* < 0.001), compared to the control group.

**Conclusion:**

The EBP education program was effective in improving the knowledge, skills, attitudes, competencies, and future use of EBP among nursing students. Hence, we recommend the EBP education program as a general education course for undergraduate nursing students to promote needed proficiency in EBP.

## Background

Evidence-based practice (EBP), the integration of the best available research with clinical expertise in the context of patient characteristics, culture, and preferences [1, 2], has been considered an effective strategy for improving the quality of care [3]. EBP represents a new paradigm in nursing; recent advances in clinical practice and research methodology as well as new information technologies have made implementation of evidence-based decision-making both feasible and desirable for nursing practice [4]. While many EBPs have been developed in the healthcare field, there has been large knowledge gaps regarding how to systematically move EBPs into usual care [5]. Likewise, adoption of EBP in nursing has been slow, and practicing nurses lack basic EBP knowledge and skills [6]. In addition, nurses in Korea still habitually overestimate and utilize evidence such as empirical knowledge or personal information resources, rather than research results, to make decisions or solve uncertainties in clinical settings. In domestic and overseas research, the actual application of the evidence-based practice in the field of practice is 20.8–37.0% [7, 8].

Facilitating factors for EBP of clinical nurses, identified as knowledge and skills, attitudes, belief in EBP, and atmosphere for EBP implementation in previous studies [9–11], could be enhanced through a well-designed EBP education program for nurses [5, 12]. A general consensus expressed in nursing pedagogy literature is the belief that the key to ensuring EBP in clinical settings lies in instilling a commitment to EBP in future nurses while they are still in nursing school [4]. EBP has been emphasized as the core competency of undergraduate nursing students and needs to be taught during simulated clinical experiences before graduation. Diffusion of the EBP competencies across nursing educational programs is a major area of attention for educators who are effecting students’ learning experiences and outcomes based on these competencies [13]. but there are few nursing colleges in Korea having an EBP course tailored for nursing undergraduate students [14].

To equip undergraduate students with basic EBP competencies, nurse educators are faced with the challenge of creating innovative and effective teaching strategies [5, 15, 16]. The documentation on EBP education mainly relates to teaching interventions among medical students and physicians [17]. There has been little published research on EBP teaching interventions among undergraduate nursing students [18]. According to a systematic review by Young et al. [17], for undergraduate students, findings from nine randomized controlled trials (RCT) indicated that a multifaceted intervention – which included various combinations of strategies such as lectures, computer lab sessions, small-group discussions, journal clubs, use of real clinical issues, portfolios and assignments – presented over a few weeks was more likely to improve knowledge, skills and attitudes when compared to a single intervention offered over a short duration, or to no intervention. Therefore, a multifaceted intervention will be necessary for effective EBP education for undergraduate nursing students. In addition, it is necessary to develop a program that can adequately handle the concepts and methodology of EBP in individual subjects in the undergraduate nursing curriculum. This will prepare undergraduate nursing students with EBP competency before clinical practicum, and then support them to apply EBP during clinical practice.

Based on this background, this study aimed to develop an EBP education program for undergraduate nursing students that used multifaceted interventions on EBP knowledge, skills, attitudes, future use of EBP, and critical thinking, and test its effects on their preparedness for EBP application to clinical practice.

## Methods

### Study design

This study used a quasi-experimental study design with pre-test, intervention, and post-test measurements undertaken immediately and 6 weeks after the intervention, to examine the effects of the EBP education program for undergraduate nursing students.

### Samples and setting

It takes four years for bachelor of nursing in Korea. The study subjects comprised the 44 students enrolled in the fourth year of their nursing degree at a college in Korea. The inclusion criteria were 1) previous enrollment in a nursing research course, 2) no enrollment in an EBP course, and 3) agreement to participate in the study. Students were reminded that participation was voluntary and that it had no relevance to their course grade. The sample size was estimated using the G*Power analysis program for repeated-measures ANOVA: within-between interactions [19]. The sample size was determined to be 42 students for both groups for repeated measures, at a significance level of .05, correlation of .50, effect size of .20, and power of 80%. The effect size was based on a study of the EBP teaching program [20]. To compensate for dropouts, we recruited 47 students (experimental group: 23; control group: 24). Of the 47 students, 1 student did not complete the intervention in the experimental group (dropout rate: 4.5%) and 2 students did not complete the survey (dropout rate: 9.1%) in the control group. Therefore, the final sample consisted of 44 students (experimental group: 22; control group: 22).

### Ethical considerations

The Institutional Review Board at Gyeongsang National University in Korea sanctioned the ethical approval for this study (Approval No. GIRB-G13-X-0021). Written informed consent was obtained from all students who agreed to participate in the study. The students, whose participation was completely voluntary, received both oral and written information about the purpose, content, and extent of the study, and were assured that their responses were confidential. Participants’ confidentiality was protected by providing a code number for each participant at the stage of data collection and analysis. In addition, the collected questionnaires were kept in a locked cabinet. After the study was completed, all questionnaires were destroyed.

The participants were informed that they had the right not to participate and could withdraw from the study at any time. The procedure of the data collection process was explained to all participants, and information regarding the estimated time and number of contacts with participants was provided. The participants were not subject to any physical, psychological, social, or economic harm or risk, as the data collection process primarily relied on a descriptive, noninvasive questionnaire. All participants were given rewards for their engagement.

### EBP education program for undergraduate nursing students (EBP-EPUNS)

The 20-h program, spread over 4 weeks and 8 sessions, was delivered to fourth year students in Korea (Table 1). The program consisted of a five-step EBP based on the study of Sackett et al. [2]: step 1 “asking clinical questions,” step 2 “evidence search.” step 3 “critical appraisal,” step 4 “implementation,” and step 5 “evaluation,” Steps 1–3, corresponding to theoretical steps, are focused on applying knowledge and skills to a clinical scenario, and steps 4–5, corresponding to practical application and evaluation, are based on the recommendation. To evaluate students’ ability to apply to clinical practice, it was decided to plan for practical application and evaluation after learning steps 1–3. It is thought that the ability to investigate problems and raise clinical questions should be prioritized to promote the EBP of undergraduate nursing students. In step 3 “critical appraisal,” the method of quality appraisal was taught and practiced only in the experimental study, providing the basis for the clinical question attempting to find effective nursing intervention, the most common clinical question in the nursing field. Although systematic review of literature is the main source providing evidence, it was excluded from the quality evaluation education because it was deemed that the nursing students had a high level of understanding [21].

**Table 1:** EBP Education Program for Undergraduate Nursing Students (EBP-EPUNS) using multifaceted interventions

Session	EBP steps	Content	Educational method	Time (minutes)
1	Introduction of EBP Clinical question (1)	Introduction of EBP	Lecture	60
		Asking clinical question (1)	Lecture	30
		Practice of asking clinical question	Group discussion Presentation	90
2	Clinical question (2)	Asking clinical question (2) Case presentation of asking clinical question	Lecture	40
		Practice of asking clinical question with scenario module	Group discussion Presentation	80
3	Evidence search (1)	Database for evidence search Using keywords and search tools	Lecture Group discussion	60
		Practice of evidence search on a computer	Computer lab session	60
4	Evidence search (2)	Hierarchy of evidence Process of evidence search Case presentation of evidence search	Lecture Group discussion	90
		Practice of evidence search with scenario module	Computer lab session Presentation	90
5	Critical appraisal (1)	Components of critical appraisal Critical appraisal of randomized controlled trials (RCTs) Level of evidence Case presentation of critical appraisal of RCTs Case presentation of grading the level of evidence	Lecture Group discussion	90
		Practice of critical appraisal of RCTs with a scenario module Practice of grading the level of evidence with scenario module	Group discussion Presentation	90
6	Critical appraisal (2)	Grading the strength of recommendations Case presentation of making recommendations	Lecture	30
		Practice of critical appraisal of RCT with a scenario module Practice of grading the level of evidence with a scenario module Practice of making recommendations and grading the strength of recommendations with a scenario module	Group discussion Presentation	90
7	Implementation and evaluation	Implementation of recommendations Evaluation of EBP process and outcomes Case presentation with implementation and evaluation	Lecture	60
		Practice of planning of implementation and evaluation with scenario module	Group discussion	120
8	Integrative application of 5-step EBP	Final practice of EBP 5-step application using a standardized patient	Group discussion Presentation	120

The researchers developed four training modules for this program: a pediatric fever module, an infant massage module, a perineal care module, and a menstrual pain module. To develop the modules, researchers first collected more than 100 clinical questions from the literature, and selected interesting topics for students. To facilitate the students’ quality appraisal, the researchers conducted a preliminary search for randomized controlled trials (RCT) of interesting topics in Korea. Then, the researchers selected the topics with several RCTs, and issued clinical questions on the selected topics in the form of Population, Intervention, Comparison, and Outcome (PICO). Finally, the researchers developed the clinical scenario and developed the training module in accordance with the clinical question. The clinical scenario in the module was pilot-tested by the researcher (KIM JS) with 10 students to ensure the relevancy, applicability, and timeliness of the developed module. The scenario was easy to apply and was easily understood by the students, and no problems were encountered during its application.

To facilitate the understanding of the 5 steps of the EBP, they were organized into two sessions, and the lectures and practice were combined. The lecture was conducted by the first author (with over 9 years of teaching experience in nursing and taking Evidence-Based Practice for 3 credits in doctoral course) in a classroom of 23 students in the experimental group. For practice, subjects were divided into 4 groups of 5–6 persons for small group practice and discussion. After the small group discussion, one of the members from each team presented her group’s discussion. At the end of all the small group presentations, the entire group discussed the contents of the oral presentation, and the researcher completed the practice session with feedback.

The content validity of the program was assessed by a nursing scholar, one of the cofounders of the Korean Society of Evidence-Based Nursing, and a nationally recognized expert in Korea with substantial knowledge of EBP methodology and experience in EBP projects.

### Data collection tool

The Outcome variables were knowledge of EBP, attitudes toward EBP, EBP skills, competencies for EBP, future use of EBP, and critical thinking. In addition, satisfaction with and understanding of the program were measured. The validity of the given questionnaire was confirmed by a panel of experts in nursing education and evidence-based practice in nursing from the original articles [22–25] that developed the scales. The reliability of the questionnaire was calculated using the alpha coefficient of internal consistency (Cronbach's alpha coefficient) from all participants (*N* = 44).

Knowledge of EBP and attitudes toward EBP were measured using an Evidence Based Practice Evaluation Competence Questionnaire (EBP-COQ), a Knowledge of EBP subscale (six items, “EBP Knowledge” hereafter), and an Attitudes toward EBP subscale (thirteen items, “EBP Attitudes” hereafter) [22]. Both subscales use 5-point Likert scales, ranging from one (strongly disagree) to five (strongly agree). Cronbach’s alpha coefficient in this study was 0.79 for EBP Knowledge and 0.74 for EBP Attitudes.

EBP Skills were measured using Cognitive Skills of Evidence-Based Practice (Lewis, Williams, & Olds, 2011) in a clinical scenario and were based on the following: ability to ask clinical questions (1 item), search for evidence (4 items), and critical appraisal (4 items). Cronbach’s alpha coefficient in this study was 0.79.

Competencies for EBP (12 items measured on a 5-point Likert scale; “EBP Competencies” hereafter) were measured using Essential Competencies for Evidence-Based Practice in Nursing [23]. Cronbach’s alpha coefficient in this study was 0.90.

Future Use of EBP was measured using a modified Future Use of EBP subscale (7 items measured on a 5-point Likert scale) of the Knowledge, Attitude and Behavior Questionnaire (KABQ) [24]. One item of “How easy or difficult has it been for you to practice evidence-based medicine as a medical student in the last month?” was deleted because of inadequacy of item content, and the Likert scale was changed from a 6-point to a 5-point scale ranging from one (strongly disagree) to five (strongly agree). Cronbach’s alpha coefficient in this study was 0.88.

Critical Thinking (35 items measured on a 5-point Likert scale) was measured using the Critical Thinking Disposition Scale for Nursing Students developed in Korea [25]. Cronbach’s alpha coefficient in this study was 0.92.

In addition to the outcome measures, the evaluation of the EBP-EPUNS was designed to assess satisfaction (5 items measured on a 5-point Likert scale) and understanding (6 items measured on a 5-point Likert scale).

### Data collection procedure

Students who agreed to participate in the study were asked to complete the demographic data and outcome variables (EBP Knowledge, EBP Attitudes, EBP Competencies, Future use of EBP, and Critical thinking) in the pretest conducted during the meeting in which they were enrolled in the study. However, EBP skills among outcome variables were not measured in the pretest. Because the instrument objectively evaluate the EBP skills of entry-level health professionals with a presentation of a clinical scenario, it is expected that participants did not have an EBP skills before EBP education.

The 4-week, 8-session, 20-h EBP education program as an intervention took place in the experimental group. To control treatment diffusion, subjects in the experimental group were asked not to share the contents of the program with the subjects of the control group.

All outcome variables were measured again, immediately and 6 weeks after the intervention, as a post-test. Satisfaction with and understanding of the program were measured immediately after intervention.

### Data analysis

SPSS software version 18.0 was used to analyze the data. Descriptive statistics were used to describe the characteristics of participants. Data were assessed for normality using the Shapiro-Wilk test. Comparative data which were not statistically different from normality were analyzed using an independent t-test and repeated measures analysis of variance (ANOVA).

A Chi-square test, Fisher’s exact test, and an independent t-test were used to compare the baseline characteristics and outcome variables between the experimental and the control groups. A repeated measures ANOVA was used to evaluate the effects of the intervention on the outcome variables. Repeated measures ANOVA is often used when data are collected three or more times in many studies with randomized controlled trials [26]. F-statistics would be computed to test for between-subjects effect (i.e., differences between experimental and controls), within-subjects effect (time factor), and treatment x time interaction effect (treatment effect to assess whether group differences varied across time) [26]. A value of *p* < 0.05 was considered statistically significant.

## Results

Baseline characteristics of the participants and results of the homogeneity tests are presented in Table 2. All 44 participants were women and the mean age of the experimental group and control group was 22.82 and 23.18, respectively. Almost all participants perceived their satisfaction with nursing as a major as neutral or above. No significant differences were found between the two groups in baseline characteristics. In addition, no group differences were found on any of the outcome variables.

**Table 2:** Characteristics of participants and homogeneity tests between the experimental and control groups (*N* = 42)

Variables	Characteristics	Experimental Group (n = 22) n (%)	Control Group (*n* = 22) n (%)	χ^2^ or t	*p* value
Gender	Female	22 (100.0)	22 (100.0)	–	–
Age (yr)	≤24	22 (100.0)	20 (90.9)	–	0.488
	≥25	0	2 (9.1)		
	Mean ± SD	22.82 ± 0.40	23.18 ± 0.85		
Religion	Yes	7 (31.8)	10 (45.5)	0.86	0.353
	No	15 (68.2)	12 (54.5)		
Satisfaction with nursing major	Very satisfied	1 (4.5)	1 (4.5)	5.21^a^	0.090
	Satisfied	15 (68.2)	8 (36.4)		
	Neutral	6 (27.3)	12 (54.5)		
	Dissatisfied	0	1 (4.5)		
	Very dissatisfied	0	0		
Satisfaction with clinical practice	Very satisfied	0	1 (4.5)	6.07^a^	0.123
	Satisfied	10 (45.5)	4 (18.2)		
	Neutral	11 (50.0)	12 (54.5)		
	Dissatisfied	1 (4.5)	4 (18.2)		
	Very dissatisfied	0	1 (4.5)		
Importance of nursing research	Very important	9 (40.9)	12 (54.5)	1.55^a^	0.547
	Important	12 (54.5)	10 (45.5)		
	Neutral	1 (4.5)	0		
	Not important	0	0		
	Not very important	0	0		
Average academic performance	≤4.0	2 (9.1)	3 (13.6)	1.48^a^	0.835
	3.5 ∼ 3.99	9 (40.9)	7 (31.8)		
	3.0 ∼ 3.49	11 (50.0)	11 (50.0)		
	2.5 ∼ 2.99	0	1 (4.5)		
	< 2.5	0	0		
Outcome variables (range of score)		Mean ± SD	Mean ± SD	t	*p* value
EBP knowledge (1–5)		3.26 ± 0.40	3.27 ± 0.46	−0.12	0.907
EBP attitudes (1–5)		3.67 ± 0.36	3.79 ± 0.32	−1.15	0.256
EBP competencies (1–5)		3.06 ± 0.48	3.04 ± 0.61	0.14	0.891
Future use of EBP (1–5)		3.83 ± 0.51	3.84 ± 0.55	−0.08	0.936
Critical thinking (1–5)		3.39 ± 0.35	3.35 ± 0.49	0.33	0.744

*EBP* Evidence Based Practice

^a^ Fisher’s Exact Test

**Fig. 1:**
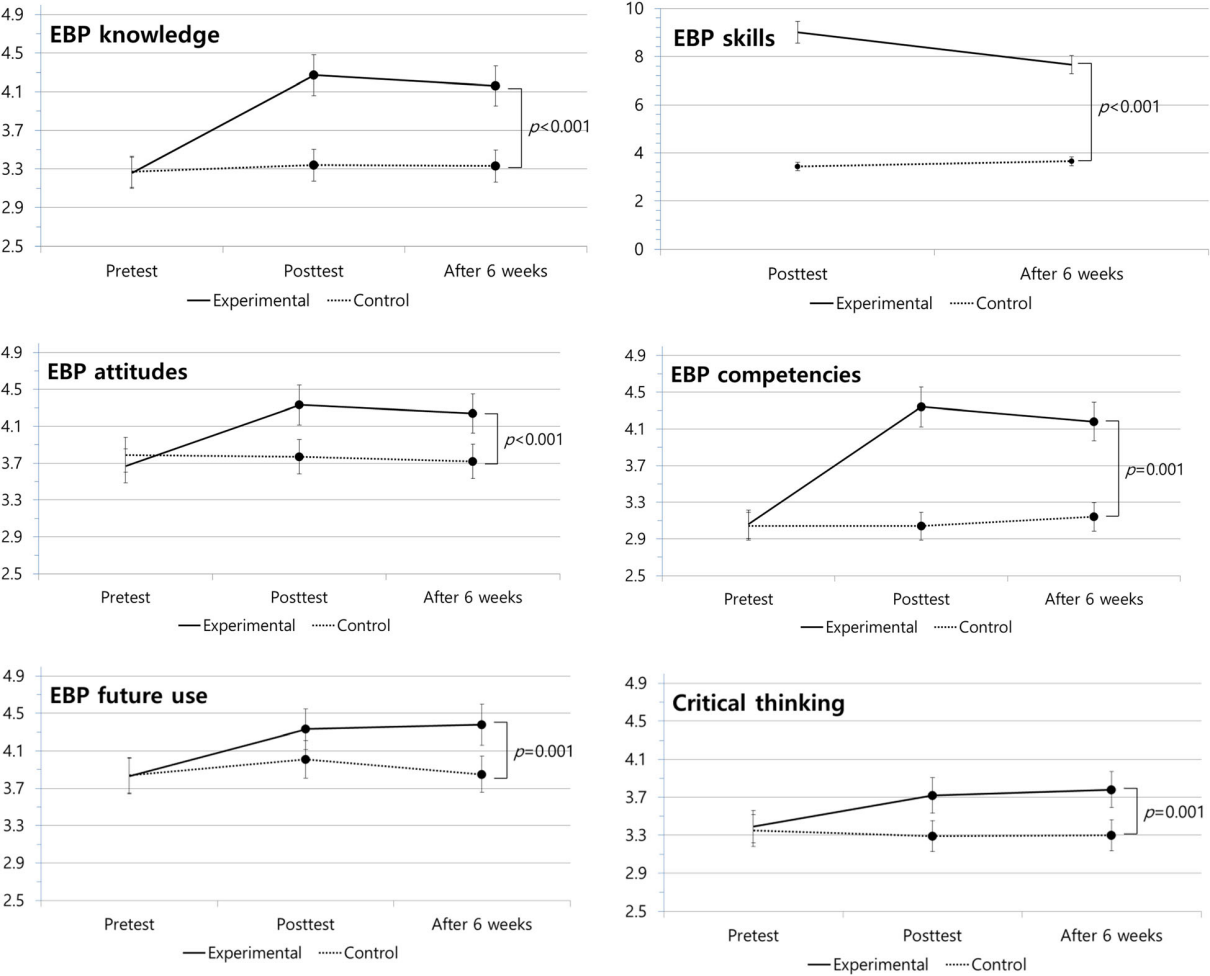
Pretest, post test, and after 6 weeks test mean (**±**SEM) of EBP knowledge, EBP skills, EBP attitudes, EBP competencies, future use for EBP and critical thinking scores for experimental (*n* = 22) and control groups (*n* = 22)

The EBP education program significantly improved all the outcomes for EBP (Table 3, Fig. 1). Using repeated measures ANOVA, we found significant interactions between group (experimental and control group) by time (from baseline to immediately and 6 weeks after the program) in EBP knowledge (F = 19.77, *p* < 0.001), EBP attitudes (F = 24.05, *p* < 0.001), EBP competencies (F = 51.47, *p* < 0.001), future use of EBP (F = 7.30, *p* = 0.001) and critical thinking (F = 17.07, *p* < 0.001).

**Table 3:** Effects of EBP education program for undergraduate nursing students

Variables	Group	Mean ± Standard Deviation			Source	F	*p* value
		Pretest	Post-test 1	Post-test 2			
		Before the program	Immediately after program	6 weeks after program			
EBP Knowledge	Experimental	3.26 ± 0.40	4.27 ± 0.51	4.16 ± 0.46	Group	22.35	< 0.001
	Control	3.27 ± 0.46	3.34 ± 0.55	3.33 ± 0.67	Time	25.56	<.0001
					Group x Time	19.77	< 0.001
EBP skills	Experimental		9.01 ± 1.52	7.66 ± 1.62			
	Control		3.44 ± 1.45	3.65 ± 1.63			
	t		12.45	8.02			
	*p* value		<.001	<.001			
EBP attitudes	Experimental	3.67 ± 0.36	4.33 ± 0.42	4.24 ± 0.42	Group	12.61	0.001
	Control	3.79 ± 0.32	3.77 ± 0.34	3.72 ± 0.34	Time	18.95	< 0.001
					Group x Time	24.05	< 0.001
EBP competencies	Experimental	3.06 ± 0.48	4.34 ± 0.37	4.18 ± 0.36	Group	39.12	< 0.001
	Control	3.04 ± 0.61	3.04 ± 0.51	3.14 ± 0.55	Time	58.59	< 0.001
					Group x Time	51.47	< 0.001
Future use of EBP	Experimental	3.83 ± 0.51	4.33 ± 0.44	4.38 ± 0.47	Group	4.86	0.033
	Control	3.84 ± 0.55	4.01 ± 0.56	3.85 ± 0.46	Time	12.39	< 0.001
					Group x Time	7.30	0.001
Critical thinking	Experimental	3.39 ± 0.35	3.72 ± 0.36	3.78 ± 0.37	Group	7.52	0.009
	Control	3.35 ± 0.49	3.29 ± 0.42	3.30 ± 0.47	Time	9.24	< 0.001
					Group x Time	17.07	< 0.001

EBP skills were not measured as a pretest. Therefore, we evaluated the effect of the program on EBP skills using an independent t-test. The results showed a statistically significant difference between the two groups immediately (t = 12.45, *p* < 0.001) and 6 weeks (t = 8.02, *p* < 0.001) after the program.

The mean score of overall satisfaction with the EBP education program was 4.45 in the 5-point Likert scale. In each item analysis, the mean score of the satisfaction scale with lecture class, practice, educator, and environment was 4.55, 4.41, 4.68, and 4.36 respectively.

The mean score of general understanding of the program was 4.0 points, out of a 5-point Likert scale. The mean score of each item were: asking clinical questions (4.41), evidence search (4.32), critical appraisal (4.00), implementation of EBP (3.86), and evaluation EBP (3.86).

## Discussion

This study aimed to develop an EBP education program for undergraduate nursing students that used multifaceted interventions on EBP knowledge, skills, attitudes, competencies, future use of EBP, and critical thinking, and test its effects on their preparedness for EBP application to clinical practice.

With the increasing emphasis on evidence-based nursing practice, nurse educators need to more fully implement teaching strategies that help students gain critical thinking skills related to inquiry and understanding of evidence-based nursing practice [27].

In a systematic review of 20 studies by Kyriakoulis et al. [18], of which 16 were pre-post studies and four were controlled trials, it was found that a majority of the studies assessed the effect of the intervention soon after the delivery of the intervention, and only 2 studies examined the longer-term effect. For reducing these methodological limitations of the previous studies, the current study included a control group and examined the long-term effect over 6 weeks following the program, as well as immediately after the program.

The EBP-EPUNS developed in this study consisted of 20 h for lecture and practice of 5 steps of EBP, through 8 sessions spread across 4 weeks. Practice of each step was done through application to a clinical scenario in an educational module developed by the researchers. The program was intended to enhance the EBP competencies of undergraduate nursing students before clinical practicum and then support them in their application of EBP to clinical practice.

The current study demonstrated that EBP-EPUNS was effective in improving the knowledge, skills, attitude, and future use of EBP of undergraduate nursing students. These findings are consistent with the findings of Ruzafa-Martinez et al. [22] that knowledge, attitudes, skills, and competencies levels of EBP were significantly improved after a 15-week EBP course for undergraduate nursing students in class. EBP skills in this study were measured with an instrument that objectively evaluated the EBP skills (skills of asking a clinical question, searching for evidence, and critical appraisal) of health professionals in a clinical scenario; thus, EBP-EPUNS was demonstrated to be effective in improving EBP skills objectively compared to the findings of Ruzafa-Martinez et al. [22], which measured EBP skills using a self-report scale.

Our findings are partly consistent with previous studies on EBP education incorporated into clinical practicum. Zhang et al. [28] found improvements in EBP knowledge, attitude, and behavior (use) following a 4-week self-directed learning and three workshops during clinical practicum. Kim et al. [29] reported improvements in EBP knowledge, but no improvements in EBP attitude and future use following EBP-focused interactive teaching interventions that consisted of a 2-h introductory lesson on the basic EBP principles and processes, as well as clinically integrated EBP group projects. These differences may result from different interventions or different educational settings, but it is possible that attitudes tend to be deep-seated and thus change slowly [29].

EBP-EPUNS was also demonstrated to be effective in improving EBP competencies among undergraduate nursing students. EBP competencies can be defined as a construct that incorporates knowledge, skills, and behavior [30], and the increase in EBP competencies in this study is considered to be the result of the improvement of the students’ knowledge, skills, and attitudes.

In addition, EBP-EPUNS was demonstrated to be effective in improving critical thinking among undergraduate nursing students. We have not found any previous experimental study in undergraduate nursing students that measured critical thinking as an outcome variable of EBP education with which to compare our findings. However, previous studies that critical thinking was significantly correlated with research utilization [31] and EBP knowledge/skills [32] indirectly support the results of this study. In this study, after a lecture on each step of EBP, a small group discussion was held in every session to apply the EBP step to the clinical scenario presented in the education module. During this time, a classroom atmosphere where every student felt free to discuss and share their experience was established. By providing two sets of encouraging questions and the opportunity of individual oral presentation for students, we proposed to clarify thinking, and reorganize their class experience with critical thinking.

We noted that this program has had lasting effects over 6 weeks after completion of intervention on all the EBP variables and critical thinking. These long-term effects could be attributed to the reflective classes. For the students in the experimental group, they practiced applying all the evidence-based practical steps based on the clinical scenarios. EBP is an important tool to make undergraduate nursing students more reflective in terms of its practical execution [33]. Reflection is found to be a vehicle that assists nursing students to analyze where they are in terms of their practice development, and identify the areas they need to further develop [34]. To be able to reflect relatedness to nursing practice, the EBP must be learned as skills that are highly valued in nursing, including critical thinking, problem solving, and self-assessment [34–36].

As for the evaluation of the program, overall satisfaction (range: 1–5 points) of the contents and composition of the program was 4.45, indicating that the participants’ program satisfaction was very high. Overall understanding (range: 1–5 points) of the contents of the program was 4.0. The reason for the high understanding level is that the practice of applying the contents of the lesson learned every hour to the clinical scenario in the education module was considered helpful.

EBP-EPUNS focused on all five steps of EBP [37]: step 1 “asking a clinical question,” step 2 “searching for the evidence,” step 3 “critical appraisal,” step 4 “implementation involving integrating the critical appraisal,” and step 5 “evaluation,” although the implementation and evaluation steps were assigned as a work plan using the clinical scenario of the educational module. Previous EBP education for undergraduate nursing students focused on 1–3 steps [22, 28], or 1–4 steps [29]. However, in case EBP education is not integrated to clinical practicum, it is not possible to practice 4–5 steps in a real clinical situation. Hence, it is more appropriate to apply these 2 steps to a clinical scenario for students instead of not including these steps in the learning of the EBP methodology.

Our teaching methodology was a multifaceted intervention that contained lectures, computer lab sessions, practice (application to clinical scenario and standardized patient case), small-group discussions, a full discussion and oral presentation, and feedback from colleagues and teachers. Systematic review showed that multifaceted interventions have been demonstrated to be more likely to improve knowledge, skills, and attitudes when compared to a single intervention offered over a short duration, or to no intervention [17]. As described above, the EBP-EPUNS for the undergraduate nursing students developed in this study was an effective program for improving EBP knowledge, skills, attitudes, competencies, future use of EBP, and critical thinking. Therefore, EBP-EPUNS is recommended to prepare for EBP competency of undergraduate nursing students before clinical practicum and then to support them to apply EBP to clinical practice.

Translating research-based knowledge into clinical practice is often hampered [38]. The EBP education program could be considered a promising strategy for congruence between the academic and practical area by establishing a collaboration between nursing teachers and practicing nurses. Researchers should think of our role in preparing our nursing students for their future role. Healthcare services demand knowledgeable and confident nurses who can work independently and take an active role in adapting EBP to meet the needs of patients. The EBP paradigm needs to be communicated, discussed, and implemented by all involved in the undergraduate nursing program. Without the spirit of cooperation and shared undertaking, implementation of EBP within the context of caring for and preparing nursing students will never materialize. The inclusion of the EBP program in the curriculum for an undergraduate nursing degree will promote the ability of students to develop skills related to the use of the EBP process, a competency that is indicated as one of the core competencies that all health professionals should develop and maintain throughout their professional career.

Subjects of this study were students who were in the fourth year of an undergraduate nursing degree with previous enrollment in a nursing research course. Findings of a systematic review by Kyriakoulis et al. [18] showed inconclusive evidence regarding the best possible time for EBP introduction into the undergraduate nursing curriculum. However, students learn about different research designs and how to match the best research design to the clinical question under consideration, and relevant statistics are also included in critiquing skills [39]. Thus, introduction to an independent EBP course in the curriculum will be appropriate after taking up nursing research in the third or fourth year of the undergraduate nursing program for nursing students.

Further research is required to examine the long-term effect of EBP-EPUNS and the effect of EBP-EPUNS on application to clinical practice. Future research to evaluate other learning outcomes such as clinical decision-making and patient outcomes is also warranted. Furthermore, when designing future EBP interventions, nurse educators should consider trends in nursing education, such as online learning or mobile device learning.

There were several limitations to this study. One was the non-randomized design of the study; however, no differences in demographic or educational data or in EBP variables were found between the groups at baseline. The other was the use of a self-report scale to measure EBP variables except EBP skills. A self-report scale may not exactly assess students’ EBP variables, and therefore it can overestimate the effect of EBP-EPUNS.

## Conclusion

EBP is the judicious use of the best current evidence in making decisions about the care of individual patients, and has been linked to the promotion of individualized care, application of best practices, and assurance of quality of nursing care. We found that the EBP-EPUNS developed in this study was effective in improving knowledge, skills, attitudes, competencies, future use of EBP, and critical thinking among undergraduate nursing students. Hence, we recommend EBP-EPUNS with multifaceted interventions as a regular course in the undergraduate nursing curriculum.

## Abbreviations

EBP: Evidence-based practice
EBP-EPUNS: Evidence-based practice education program for undergraduate nursing students
SEM: Standard error of mean
